# Retraction: The Cellular Distribution of Serotonin Transporter Is Impeded on Serotonin-Altered Vimentin Network

**DOI:** 10.1371/journal.pone.0211966

**Published:** 2019-02-01

**Authors:** The PLOS ONE Editors

The corresponding author of this article [1] notified the journal that some figures are problematic and the original data underlying the reported results are no longer available.

There are similarities between data shown within and across a number of Western blot figures, as listed below. In the following list, semicolons are used to separate different groups of similar data, and commas are used in listing bands within a similarity group. Similarities for a clustered group of lanes indicate blocks of similarity, e.g. similarities noted between lanes 1–3 and lanes 4–6 of a blot would mean that lane 1 is similar to lane 4, lane 2 is similar to lane 5, and so on.

Fig 2:

- Similarities between Fig 2B lanes 1 and 2
- Similarities between Fig 2D lanes 1, 2, 3
- Similarities between Fig 2B lane 3, and Fig 8A CHO-SERT/SERT(surface)/pS56-Ab lane 3

Fig 5A:

- SERT-Ab blot: similarities between bands shown in lanes 1, 6; lanes 2, 8; lanes 3, 10; and lanes 4, 5
- Actin blot: similarities between lanes 2–6 and lanes 11–7, when flipped horizontally

Fig 7:

- 7A: similarities between bands shown in lanes 1, 3, 4, 7; and lanes 2, 6, 8, 9
- 7B: similarities between bands shown in lanes 3, 4, 5, 7; and lanes 2, 6, 8, 9, 10
- 7C: similarities between bands shown in lanes 5, 7, 8, 9
- 7D: similarities between bands shown in lanes 1, 3, 4, 5, 7; and lanes 2, 6, 8, 9, 10
- 7E: similarities between bands shown in lanes 1–4 and 8–5, when flipped horizontally; and between bands in lanes 2, 7, 11; lanes 3, 6, 10; and lanes 4, 5, 9
- 7F: similarities between bands shown in lanes 1, 3, 5, 7, 9, 11; and lanes 2, 4, 6, 8, 10

Fig 8A:

- Similarities between bands shown in all lanes of the lower pS56-Ab blots (CHO-(SERT+VimentinS56A), for both SERT (surface) and SERT (in))
- Similarities between lanes 2,3 of the CHO-SERT/SERT(surface)/SERT-Ab blot, and lanes 2, 1 of the CHO-(SERT+VimentinS56A)/SERT(surface)/SERT-Ab blot, when flipped horizontally

Across Figures:

- Similarities between Fig 2C lanes 1–3, and Fig 5A lanes 4–6
- Similarities between bands in Fig 4 lanes 1, 4, 5, 6, and Fig 6 lanes 1, 7
- Similarities between bands in Fig 4 lane 2, and Fig 6 lanes 2, 5
- Similarities between bands in Fig 4 lane 3, and Fig 6 lane 6
- Similarities between bands in Fig 8A, lanes 1–2 of the upper pS56-Ab blots for SERT (surface) and SERT (in), and Fig 1C lane 3

The University of Arkansas for Medical Sciences performed an internal review of this work and recommended retraction in light of concerns about the Western blot figures. The University noted that the original data are not available, and that the conclusions of the article are not supported by available data.

In light of these issues, and in line with the institution’s recommendation, the *PLOS ONE* Editors retract this article.

DT, BCJ, NJR, VL agreed with the retraction. FK did not agree with the retraction. JTH, ST, PZ did not respond. MF, BAA, EZ, IAB could not be reached.
